# Using Computed Tomography Scans and Patient Demographic Data to Estimate Thoracic Epidural Space Depth

**DOI:** 10.1155/2015/470240

**Published:** 2015-04-16

**Authors:** Alyssa Kosturakis, Jose Soliz, Jackson Su, Juan P. Cata, Lei Feng, Nusrat Harun, Ashley Amsbaugh, Rodolfo Gebhardt

**Affiliations:** ^1^University of Texas San Antonio Health Science Center, San Antonio, TX 78249, USA; ^2^Department of Anesthesiology and Perioperative Medicine, The University of Texas MD Anderson Cancer Center, Houston, TX 77030, USA; ^3^Department of Biostatistics, The University of Texas MD Anderson Cancer Center, Houston, TX 77030, USA; ^4^Department of Anesthesiology and Perioperative Medicine, University of Louisville, Louisville, KY 40292, USA; ^5^Department of Pain Medicine, The University of Texas MD Anderson Cancer Center, Houston, TX 77030, USA

## Abstract

*Background and Objectives*. Previous studies have used varying methods to estimate the depth of the epidural space prior to placement of an epidural catheter. We aim to use computed tomography scans, patient demographics, and vertebral level to estimate the depth of the loss of resistance for placement of thoracic epidural catheters.* Methods*. The records of consecutive patients who received a thoracic epidural catheter were reviewed. Patient demographics, epidural placement site, and technique were collected. Preoperative computed tomography scans were reviewed to measure the skin to epidural space distance. Linear regression was used for a multivariate analysis.* Results*. The records of 218 patients were reviewed. The mean loss of resistance measurement was significantly larger than the mean computed tomography epidural space depth measurement by 0.79 cm (*p* < 0.001). Our final multivariate model, adjusted for demographic and epidural technique, showed a positive correlation between the loss of resistance and the computed tomography epidural space depth measurement (*R* ^2^ = 0.5692, *p* < 0.0001).* Conclusions*. The measured loss of resistance is positively correlated with the computed tomography epidural space depth measurement and patient demographics. For patients undergoing thoracic or abdominal surgery, estimating the loss of resistance can be a valuable tool.

## 1. Introduction

Epidural analgesia is widely used for postoperative pain management because of the advantages that epidural analgesia offers in reducing postoperative pain and the surgical stress response [1–3]. Correct placement of an epidural catheter into the epidural space using the loss of resistance (LOR) technique is a difficult skill to teach because it is performed “blind” and anatomical variations can lead to the identification of false LOR. One study showed that less than half of resident trainees were able to reach acceptable failure rates after performing 21 epidurals [4]. Failure to correctly identify the epidural space may result in a variety of undesirable outcomes, such as failure to provide effective analgesia, dural puncture, or spinal cord injury.

A number of studies have measured the mean skin to epidural space depth (SES) in various patient populations and have proposed prediction models based on anthropometric measures and imaging. Carnie et al. showed that computed tomography (CT) was a useful tool to predict SES; however, estimation of SES in this model is dependent on the angle of needle insertion, which would not be known prior to epidural placement [5]. Kao et al. also proposed a CT-based prediction model for epidural placement at the T10-T11 interspace using the paramedian approach, but the clinical relevance of this model is minimized by the fact that the model assumes a constant angle of needle insertion [6].

SES depth at the lumbar intervertebral level has also been studied in the obstetric patient population [7–9]. However, changes in the epidural space that occur during pregnancy, as well as differences between the morphology of the thoracic and lumbar regions of the spine, make these studies inapplicable to nonobstetric patients receiving an epidural at the thoracic intervertebral levels. The caudally angled spinous processes at the thoracic vertebral level make placement of a thoracic epidural more challenging.

The objective of the current study was to evaluate the utility of CT in predicting SES as measured by the LOR technique and to use CT scan measurements in conjunction with patient demographic and anthropometric data (height, age, weight, body mass index (BMI), and ethnicity) to create a multivariate prediction model of SES in nonobstetric patients with cancer undergoing thoracic or open abdominal surgery.

## 2. Materials and Methods

Following approval from The University of Texas MD Anderson Cancer Center Institutional Review Board, we retrospectively reviewed the records of patients who had epidurals placed between the T3 and T12 intervertebral levels by 1 of 2 providers at MD Anderson between July 1, 2007, and June 30, 2011. We noted demographic and anthropometric data for each patient, including ethnicity, age, weight, height, and BMI, at the time the procedure was performed. We also noted the epidural placement approach (midline or paramedian) and the LOR to the nearest half centimeter as reported by the attending anesthesiologist performing the epidural.

In all cases a Tuohy needle with 1 cm markings was used to insert the epidural catheter into the epidural space. Records were excluded from our analysis if the approach or LOR measurement was not documented or if the epidural was attempted but no catheter was placed. A blinded anesthesiologist reviewed CT scans completed within 1 month prior to the patient receiving the epidural and measured the SES. The SES was measured using an internal digital measuring tool (iSite PACS, Philips, Amsterdam, Netherlands), which reported measurements accurate up to 1 hundredth of a centimeter. If the epidural was placed using the midline approach, the SES was defined as the perpendicular length from the skin to the ligamentum flavum. If the paramedian approach was used, SES was defined as the segment located 1 cm lateral from the midline to the ligamentum flavum.

BLiP plots were used to display the distributions of reported measurements of LOR and SES as determined from the CT images (SES-CT). The Spearman rank correlation coefficient was used to analyze the degree of correlation between LOR and SES-CT measurements and associations between demographic or anthropometric factors and LOR or SES-CT measurements. The Wilcoxon rank-sum test was used to assess associations between categorical variables and LOR or SES-CT measurements. The Wilcoxon signed-rank test was used to evaluate the differences between LOR and SES-CT measurements. Linear regression in a multivariate analysis was used to examine the association between LOR and SES-CT measurements, adjusted for clinical factors. A *p* value of less than 0.05 was considered statistically significant. SAS version 9.2 and S-Plus version 8.0 were used to perform all analyses.

## 3. Results

**Figure 1:**
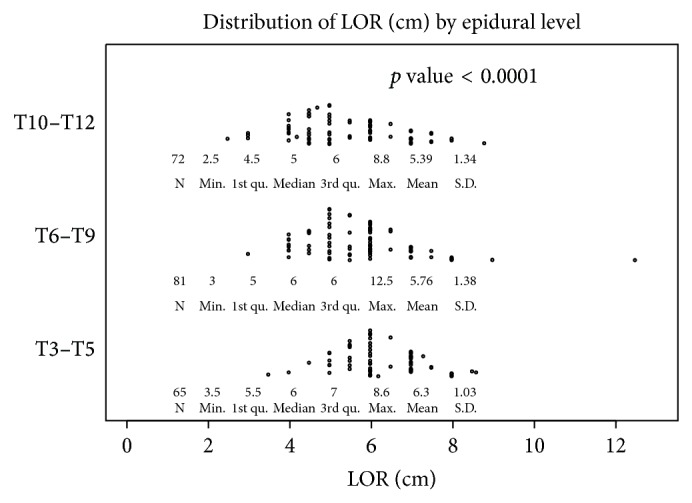
Distribution of loss of resistance (LOR) measurements (cm) for thoracic levels T3–T5, thoracic levels T6–T9, and thoracic levels T10–T12.

The records of 218 patients were reviewed for our analysis. Among these patients, 164 (75.2%) were white, 30 (13.8%) were Hispanic, 12 (5.5%) were of African descent, and 12 (5.5%) were Asian. Patient anthropometric data are shown in Table 1. Epidural placement details are shown in Table 2. The midline epidural placement approach was used in 96 patients (44.0%) and the paramedian approach was used in the remaining 122 patients (56.0%; Table 2). In 65 patients (29.8%), the epidural was placed at thoracic levels T3–T5, 81 patients (37.2%) had the epidural placed at thoracic levels T6–T9, and 72 patients (33%) had epidurals placed at thoracic levels T10–T12 (Table 2). Three separate distributions by BLiP plots are displayed for thoracic levels T3–T5, T6–T9, and T10–T12 (Figure 1). Epidural LOR measurements had an increasing trend with higher thoracic level (*p* < 0.0001).

**Figure 2:**
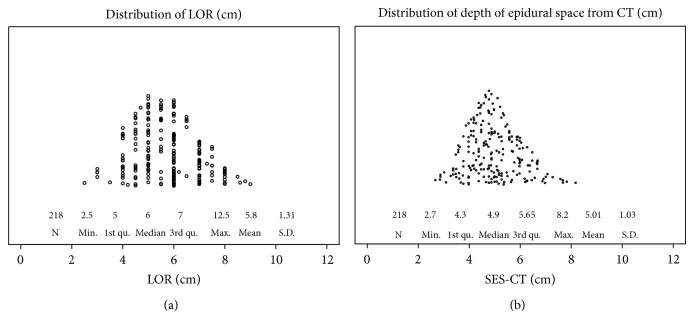
Distributions of measurements (cm) of (a) loss of resistance (LOR) and (b) skin to epidural space depth determined from computed tomography (SES-CT).

**Figure 3:**
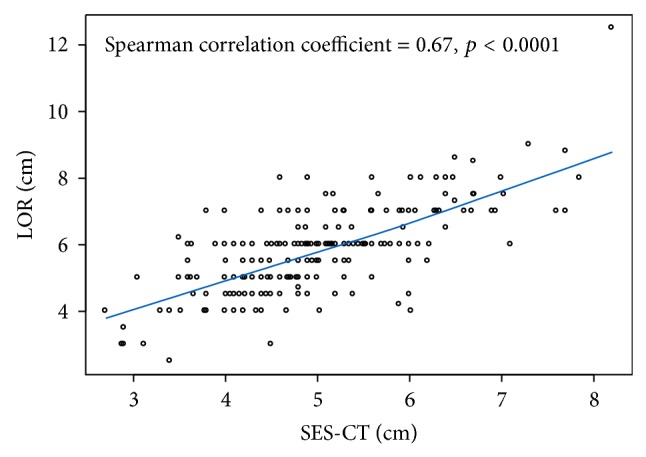
Correlation between measurements (cm) of loss of resistance (LOR) and skin to epidural space depth determined from computed tomography (SES-CT).

BLiP plots (Figure 2) show the distributions of recorded LOR (cm) and SES-CT (cm) measurements for our patient population. The mean LOR measurement was 5.80 ± 1.31 cm and the mean SES-CT measurement was 5.01 ± 1.03 cm. The mean LOR measurement was significantly larger than the mean SES-CT measurement, by 0.79 cm (Wilcoxon signed-rank test, *p* < 0.001), but the LOR and SES-CT measurements were positively correlated (Spearman correlation coefficient = 0.67, *p* < 0.0001; Figure 3). The difference between LOR and SES-CT measurements was not correlated with age (*p* = 0.553), weight (*p* = 0.973), height (*p* = 0.111), BMI (*p* = 0.186), or ethnicity (*p* = 0.225). For patients whose epidural was placed using the midline approach, the difference between LOR and SES-CT measurements was significantly larger than for those whose epidural was placed using the paramedian approach (*p* = 0.0094; Table 3).

The LOR measurements were negatively correlated with age (Spearman correlation coefficient = −0.19, *p* = 0.004) but positively correlated with BMI (Spearman correlation coefficient = 0.49, *p* < 0.0001). Asian patients had on average slightly lower LOR measurements, but the difference was not significant (*p* = 0.0612). LOR measurements did not differ between patients with epidurals placed using the midline approach and those with epidurals placed using the paramedian approach (*p* = 0.3408).

SES-CT measurements were also negatively correlated with age (Spearman correlation coefficient = −0.21, *p* = 0.002) and positively correlated with BMI (Spearman correlation coefficient = 0.72, *p* ≤ 0.0001). SES-CT measurements also differed across ethnicities (*p* = 0.0015). Asian patients had significantly lower SES-CT measurements than white, African, and Hispanic patients. Asian patients also had significantly lower BMIs than those of the other 3 ethnicities (*p* < 0.0015). SES-CT measurements did not differ between patients with epidurals placed using the midline approach and those with epidurals placed using the paramedian approach.

**Figure 4:**
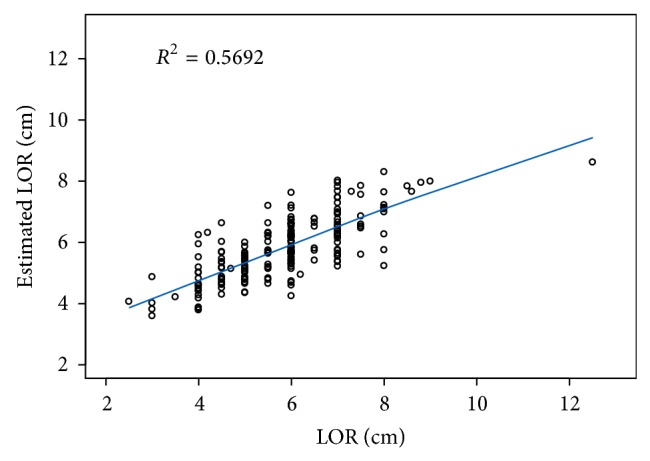
Correlation between measurements (cm) of loss of resistance (LOR) and skin to epidural space depth determined from computed tomography (SES-CT), adjusted for demographic and anthropometric factors.

Our final estimated linear regression model, with adjustments for demographic and anthropometric factors, showed a positive correlation between LOR and SES-CT measurements (*R* ^2^ = 0.5692, *p* < 0.0001) when adjusted for approach (median or paramedian) and epidural level (Figure 4). The estimated model is as follows:

(1) Estimated  LOR=0.80+0.90×SES-CT+0.19×median=1,paramedian=0+ T3–T5=0.79  or  T6–T9=0.40.

Finally to ensure that there were no provider-related effects on the data, we analyzed the measured LOR, SES-CT, epidural placement level, epidural placement approach, and difference in LOR and SES-CT between the two anesthesiologists. There were no significant differences for the two providers (all *p* > 0.05).

## 4. Discussion

Our results indicate that CT measurements can be a valuable tool for estimating SES prior to epidural placement in the cancer patient population, and we have created a multivariate prediction model that does so while taking into account demographic and anthropometric variables.

The placement of thoracic epidurals is especially challenging because of the caudally angled spinous processes in the thoracic region of the spine. Misplacement of the epidural catheter can result in a number of unintended consequences. If the epidural needle is advanced past the epidural space resulting in dural puncture, inadvertent spinal block or, in rare cases, nerve injury could result. Conversely, placement of the epidural needle outside the epidural space will not provide effective analgesia. At our institution, patients undergo diagnostic imaging, and CT or magnetic resonance imaging scans are used as part of the cancer-related evaluation. Our study is unique because it examines the impact of demographic and clinical factors as well as the use of preoperative CT scan measurements on the estimation of SES. Estimation of the LOR depth prior to inserting the needle is helpful particularly in instructing less-skilled providers (residents and fellows), whose patients may be at increased risk of complications.

Past studies have reported mean SES values in various patient populations, using various approach methods, and at various intervertebral levels. Our study focused on epidurals placed in the thoracic vertebra of cancer patients for postoperative pain control. Our data indicate that both reported LOR and SES-CT measurements were correlated with demographic and clinical variables, including weight, height, BMI, and ethnicity. This is consistent with previously reported findings from several other studies [6, 10, 11]. However, our study also focused on differences in LOR and SES-CT measurements according to epidural placement technique and the intervertebral space at which the epidural catheter was placed. Our data suggest that epidurals placed at T3–T5 had significantly larger LOR measurements than epidurals placed at T6–T12. When placing an epidural, it is often necessary to angle the needle in a more cephalad trajectory in the thoracic region owing to the caudally angled spinous processes, which may help to explain this finding.

Contrary to the findings of Carnie et al., we found that LOR measurements were significantly larger than SES-CT measurements [5]. We postulate that our result is sensible given that the supine position the patient assumes while undergoing CT compresses the subcutaneous tissues, yielding a smaller measurement than when the patient is in the sitting (head down) position for epidural placement. This is supported by the findings of Lee et al., who reported an overall greater separation of the dura from the cord in thoracic regions of the spinal cord in the sitting position compared with the supine position [12]. Because SES-CT measurements were obtained using a 2-dimensional scan, we were also unable to account for cephalad angulation of the needle during epidural placement. This is another potential explanation for the smaller SES-CT measurements compared with LOR measurements. Another factor that potentially accounted for the difference is that, when placing the epidural catheter, the anesthesiologists used surface anatomy and landmarks to estimate the thoracic level. It is possible that in practice the epidural was placed 1 vertebra higher or lower than reported in the chart [13].

As expected, we found that patient demographic characteristics did not account for the difference (0.79 cm) we observed between the reported LOR and SES-CT measurements. However, we found that the degree of correlation between SES-CT and LOR depended on whether the epidural was placed using the midline or paramedian approach and on the thoracic level at which it was placed. For epidurals placed using the paramedian approach, the correlation between LOR and SES-CT measurements was significantly better. This can be explained by the spinal morphology in the thoracic region. When a midline approach is used, the needle is usually more tilted at a cephalad angle than in the paramedian approach to compensate for the caudally angled spinous processes. In the paramedian approach, the needle enters more laterally, and less of a cephalad angle is required to reach the epidural space.

The correlations were consistent between demographic factors and LOR measurements or SES-CT measurements. For both types of measurements, BMIs were positively correlated and age was negatively correlated with the measurement. A high BMI, which is associated with high body fat levels, increases SES [14]. The decrease in body fat associated with aging may explain the negative association of aging with LOR and SES-CT measurements [15]. Only with the SES-CT measurements were we able to detect differences across the ethnicities. Asian patients had significantly lower SES-CT measurements than patients of other ethnicities, but differences in BMI across ethnicities may account for this difference.

Previous attempts at estimating LOR measurements using demographic and clinical data have demonstrated varying success. One published estimation model suggests that SES can be calculated using the Pythagorean theorem, whereby LOR = SES-CT/cos (angle of insertion) [5]. However, this model is limited because the exact angle of insertion would not be known prior to placement of the epidural. Other studies have produced multivariate prediction models for estimating SES using demographic data, epidural insertion site, and needle insertion technique in the model; however, these models do not incorporate the use of radiologic measurements [9, 11]. In our study, we produced a linear regression prediction model for estimating LOR that includes adjustments in the SES-CT value for BMI, approach (midline or paramedian), and thoracic level. This model showed that SES-CT measurements were predictive of LOR (*R* ^2^ = 0.5692, *p* < 0.0001).

Although our model provides valuable information, it may have some limitations in predicting LOR measurements in future patients because the model is representative of the 218 patients that were studied. The authors also note that this was a retrospective study; thus its results may have been influenced by confounding or unmeasured factors. In addition, the widely increasing use of ultrasound may provide additional real-time measurements that our model does not include [16–18]. A recent meta-analysis showed that using lumbar neuraxial ultrasound to guide epidural placement increased the success of neuraxial blocks and reduced the number of insertion attempts [19]. Perhaps the addition of real-time ultrasound guided measurements may strengthen our estimation model.

In conclusion, study demonstrates how demographic and clinical factors may affect thoracic epidural placement in the cancer patient population, in which postoperative pain is increasingly being managed with epidural analgesia. In addition, for patients undergoing elective thoracic or abdominal surgery that requires a preoperative CT scan, estimating LOR using SES-CT measurements can be a valuable tool.

## Figures and Tables

**Table 1:** Patient anthropometric characteristics (*n* = 218).

Variable	Mean ± standard deviation	Min.	Max.	Median
Age at procedure	55.79 ± 17.48	18	88	56
Weight, kg	82.87 ± 19.50	42.00	166.00	81.80
Height, m	1.70 ± 0.11	1.45	1.95	1.71
BMI	28.55 ± 5.96	17.70	64.84	27.95
LOR, cm	5.80 ± 1.31	2.50	12.50	6.00
SES-CT, cm	5.01 ± 1.03	2.70	8.20	4.90

BMI, body mass index; LOR, loss of resistance measurement; SES-CT, skin to epidural space depth as measured by computed tomography.

**Table 2:** Epidural placement approach and level (*n* = 218).

Category	Number (%)
Midline	96 (44.04)
Paramedian	122 (55.96)
Epidural level	
T3–T5	65 (29.8)
T6–T9	81 (37.2)
T10–T12	72 (33.0)

**Table 3:** Differences (cm) between measurements of loss of resistance (LOR) and skin to epidural space depth determined from computed tomography (SES-CT).

Variable	Number	Mean ± standard deviation	Min.	Max.	*p* value
Ethnicity					0.225
Asian	12	0.51 ± 0.74	−0.34	1.90	
African	12	0.39 ± 0.85	−1.10	1.52	
White	164	0.85 ± 0.98	−2.02	4.30	
Hispanic	30	0.73 ± 0.68	−0.70	2.20	
Epidural placement					0.0094
Midline	96	0.95 ± 0.88	−1.69	3.20	
Paramedian	122	0.67 ± 0.95	−2.02	4.30	
Epidural level					<0.0001
T3–T5	65	1.21 ± 0.86	−0.70	3.40	
T6–T9	81	0.80 ± 0.92	−1.50	4.30	
T10–T12	72	0.40 ± 0.84	−2.02	3.10	
